# Acute Rheumatic Fever: Where Do We Stand? An Epidemiological Study in Northern Italy

**DOI:** 10.3389/fmed.2021.621668

**Published:** 2021-02-24

**Authors:** Achille Marino, Rolando Cimaz, Maria Antonietta Pelagatti, Giulia Tattesi, Andrea Biondi, Laura Menni, Marco Sala, Patrizia Calzi, Francesco Morandi, Francesca Cortinovis, Anna Cogliardi, Claudia Addis, Roberto Bellù, Massimo Andreotti, Tiziana Varisco

**Affiliations:** ^1^Department of Pediatrics, Desio Hospital, Azienda Socio Sanitaria Territoriale Monza, Monza, Italy; ^2^Azienda Socio Sanitaria Territoriale G.Pini-Centro Traumatologico Ortopedico, Milan, Italy; ^3^Department of Clinical Sciences and Community Health, and Research Center for Adult and Pediatric Rheumatic Diseases, University of Milan, Milan, Italy; ^4^Department of Pediatrics, Milano-Bicocca University Monza e Brianza per il Bambino e la sua Mamma Foundation, Monza, Italy; ^5^Department of Pediatrics, Vimercate Hospital, Vimercate, Italy; ^6^Department of Pediatrics, Carate Hospital, Carate Brianza, Italy; ^7^Department of Pediatrics, San Leopoldo Mandic Hospital, ASST Lecco, Lecco, Italy; ^8^Department of Pediatrics, Lecco Hospital, ASST Lecco, Lecco, Italy

**Keywords:** acute rheumatic fever, group A β-hemolytic streptococcus, carditis, Jones criteria, penicillin

## Abstract

Acute rheumatic fever (ARF) is a non-septic complication of group A β-hemolytic streptococcal (GAS) throat infection. Since 1944, ARF diagnosis relies on the Jones criteria, which were periodically revised. The 2015 revision of Jones criteria underlines the importance of knowing the epidemiological status of its own region with updated data. This study aims to describe ARF features in a retrospective cohort retrieved over a 10-year timespan (2009–2018) and to report the annual incidence of ARF among children in the Province of Monza-Brianza, Lombardy, Italy during the same period. This is a multicentric cross-sectional/retrospective study; 70 patients (39 boys) were diagnosed with ARF. The median age at diagnosis was 8.5 years (range, 4–14.2 years). Overall, carditis represented the most reported major Jones criteria followed by arthritis and chorea (40, 27, and 20 cases, respectively). In order to calculate the annual incidence of ARF, only children resident in the Province of Monza-Brianza were included in this part of the analysis. Therefore, 47 patients aged between 5 and 14 years were identified. The median incidence during the study time was 5.7/100,000 (range, 2.8–8.3/100,000). In the Province of Monza-Brianza, we found an incidence rate of ARF among children aged 5–14 years constantly above the threshold of low-risk area as defined in the 2015 revision of Jones criteria. Therefore, the diagnosis of ARF should be based on the moderate–high-risk set of Jones criteria. However, given the burden of secondary prophylaxis, expert opinion is advisable when the diagnosis of ARF is uncertain.

## Introduction

Acute rheumatic fever (ARF) is a non-septic complication of group A β-hemolytic streptococcal (GAS) throat infection. ARF is a multisystem autoimmune disease causing inflammation in different sites (joint, heart, basal ganglia, skin); it affects typically school-aged children (particularly between 5 and 14 years) without sex differences (1).

Several factors influence the likelihood of ARF developing such as genetic predisposition and the virulence of the infecting GAS strain. It has been estimated that <3% of patients who have a GAS tonsillopharyngitis develops ARF (2). The prompt identification and treatment of GAS pharyngitis lowers the risk of this condition. ARF pathogenesis is not completely clear, and there is no sound experimental model. Nevertheless, the GAS type-specific antigen, the M protein, has been found to eventually cause, in a predisposed host, tissue inflammation through the formation of autoantibodies and the activation of cell-mediated immunity by acting as a superantigen. Furthermore, the immunological pathogenesis is supported by the typical latency of about 3 weeks between GAS pharyngitis and ARF onset (3, 4). The genetic predisposition for ARF might be acquired (44% of concordance among monozygotic twins), although this inheritance is polygenic with variable penetrance (5, 6). Joint involvement represents the most frequent clinical manifestation along with heart valvulitis, while chorea is less common (7). Heart involvement may result in permanent damage of valves resulting in rheumatic heart disease (RHD) and represents the major cause of acquired heart disease in the developing world (8, 9). The other ARF manifestations resolve without permanent consequences.

The incidence of ARF is highly variable depending on the geographic areas (10). Middle East, Asia, Eastern Europe, and Australia show a higher incidence (7.5–194 cases/100,000/year) compared to US (0.61 cases/100,000/year), even if an overall downward trend has been observed (11, 12). Since 1944, ARF diagnosis relies on the Jones criteria, which were periodically revised (13–15). The last revision of 2015 was principally driven by the different disease burden around the world and the increasing role of echocardiography. Furthermore, the growing use of over-the-counter drugs has been listed as one of the potential factor impacting clinical presentation. In the 2015 revision, patients were stratified as having low or moderate–high risk for ARF according to the local disease incidence, with cutoff for low incidence being <2 per 100,000 school-aged children (usually 5–14 years old) per year or an all-age prevalence of RHD of ≤ 1 per 1,000 per year. Therefore, two sets of criteria have been provided according to the risk of ARF (15). Furthermore, the presence of subclinical carditis was considered as a major manifestation. The 2015 revision of Jones criteria underlines the importance of knowing the epidemiological status of its own region with updated data. ARF resurgence in Italy has been suggested by several studies (1, 16, 17).

The province of Monza-Brianza is one of the 12 provinces of Lombardy, a region of northwest of Italy. Despite being the smallest province of Lombardy in terms of size (405.49 km^2^), the Province of Monza-Brianza has one of the highest population densities of the region (residents/km^2^, 2.18; population of 878,267 to 1 January 2020 from ISTAT data). This study aims to describe ARF features in a retrospective cohort retrieved over a 10-year timespan (2009–2018) and to report the annual incidence of ARF among children in the Province of Monza-Brianza, Lombardy, Italy during the same period.

## Materials and Methods

This is a multicentric cross-sectional/retrospective study involving Hospitals of the Province of Monza-Brianza (Desio, San Gerardo of Monza, Vimercate, and Carate Hospital) in addition to two nearby collaborating Hospitals (Merate and Lecco Hospital). The inclusion criteria were the diagnosis of ARF between January 1, 2009 and December 31, 2018 and a disease onset before the age of 18 years. Only hospitalized patients were included in the present study. ARF diagnosis was established according to the criteria then in place. ARF cases were first identified by analyzing hospital discharge records (HDRs) according to the International Classification of Disease, Ninth Revision, Clinical Modification (ICD-9 CM). The following ICD-9 CM codes were used: 390, 391.0, 391.1, 391.2, 391.8, 391.9, 392.0, and 392.9. A chart review of each retrieved case was then performed in order to acquire demographical and clinical data and to avoid overlaps and duplicates. All patients underwent echocardiography during the acute phase of the disease. Subclinical carditis was defined as positive echocardiography in the absence of clinical features. Details on the retrieved cohort (demographic, clinical, laboratory, and echocardiographic data) were collected in a customized database. To calculate the annual incidence of ARF in the Province of Monza-Brianza, patients not resident in this province were excluded from the analysis as well as those younger than 5 years and older than 14 years. This subgroup of patients was compared with the age-matched population of the same area of the same year. School-aged children (5–14 years) population for each year was abstracted from the National Institute of Statistics (ISTAT) (http://demo.istat.it/). A descriptive analysis was then undertaken (Excel 2011). Since anonymized data were used, according to local regulations, no ethics committee approval was deemed necessary.

## Results

Over the 10-year timespan, 70 patients (39 boys) were diagnosed with ARF. The majority were Caucasians (*n* = 60). The median age at diagnosis was 8.5 years (range, 4–14.2 years). The major features of the cohort are shown in Table 1.

**Table 1:** Acute rheumatic fever (ARF) features.

**ARF features**	**No. of patients (%)**
**Major criteria**	
Arthritis	27 (39%)
Carditis	40 (57%)
Subclinical carditis	16 (40%^*^)
Mitral valve	19 (47.5%)
Aortic valve	6 (15%)
Tricuspid valve	1 (2.5%)
Bivalvular	14 (35%)
Chorea	20 (29%)
Erythema marginatum	4 (6%)
Subcutaneous nodules	0
**Minor criteria**	
Fever	32 (46%)
Arthralgia	35 (50%)
Increased inflammatory markers	39 (56%)
Prolonged PR interval	3 (4%)

* *It refers to patients with carditis only*.

Overall, carditis represented the most reported major Jones criteria followed by arthritis and chorea (40, 27, and 20 cases, respectively). Erythema marginatum was quite rare, and in three out of four cases, it was present together with carditis. No subcutaneous nodule was recorded. Among associations of major criteria, arthritis and carditis was the most frequent (*n* = 14), followed by carditis and chorea (*n* = 10). Carditis without other major criteria was recorded 13 times, while isolated arthritis was recorded 12 times and chorea 9 times. The mitral valve was the most commonly affected heart valve, either as an isolate finding (*n* = 19) or along with aortic (*n* = 12) or tricuspid valve involvement (*n* = 2); conversely, the aortic valve was affected more rarely (*n* = 6). Subclinical carditis was identified in 16 patients.

Increased inflammatory markers were the most frequently recorded minor criterion, followed by arthralgia and fever (39, 35, and 32 cases, respectively). Prolonged PR interval was observed in three cases, two of them with associated valvulitis. Information on acute treatment was available for 48 subjects. Ibuprofen was the most used non-steroidal anti-inflammatory drug (NSAID): 17 patients received ibuprofen compared with 7 patients treated with acetylsalicylic acid. Corticosteroids were used in 28 cases. The use of corticosteroids was mainly driven by the presence of carditis (20 cases, 8 with bivalvular involvement and 8 patients with concomitant chorea) and, less frequently, to treat chorea alone (6 cases) or arthritis and erythema marginatum (one case each). Secondary prophylaxis for GAS infection with intramuscular injection of penicillin was administered in all the patients according to their weight (600,000 U for patients <27 kg, 1,200,000 U for patients >27 kg). Secondary prophylaxis was administered every 3 weeks in 36 patients and every 4 weeks in 29 patients, respectively (no information available for 5 patients).

Data about the suggested duration of secondary prophylaxis were available for 59 patients. The majority (*n* = 40) were advised to continue GAS prophylaxis until 21 years of age. In this group, 22 patients had carditis. Two patients with severe bivalvular carditis were recommended to continue secondary prophylaxis until 40 years of age and lifelong, respectively. Discontinuation of GAS prophylaxis before 21 years of age was suggested to 17 patients (10 with carditis). Indeed, four patients (all with carditis) were recommended to continue secondary prophylaxis until 18 years of age, 3 patients (all with carditis) for 10 years, and 10 patients (3 with carditis) for 5 years.

**Figure 1:**
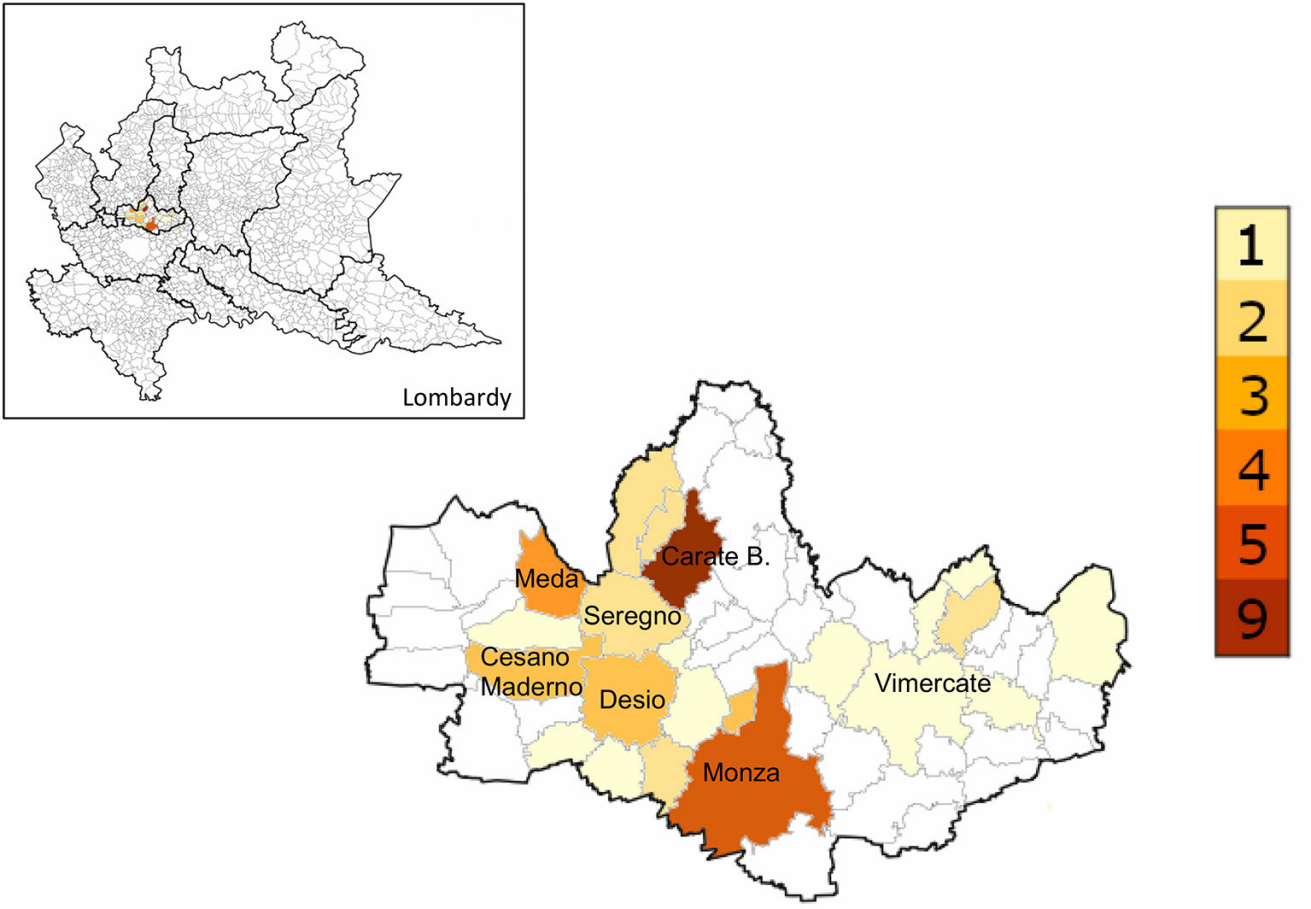
Geographic distribution of acute rheumatic fever cases in the Province of Monza-Brianza between 2009 and 2018.

**Figure 2:**
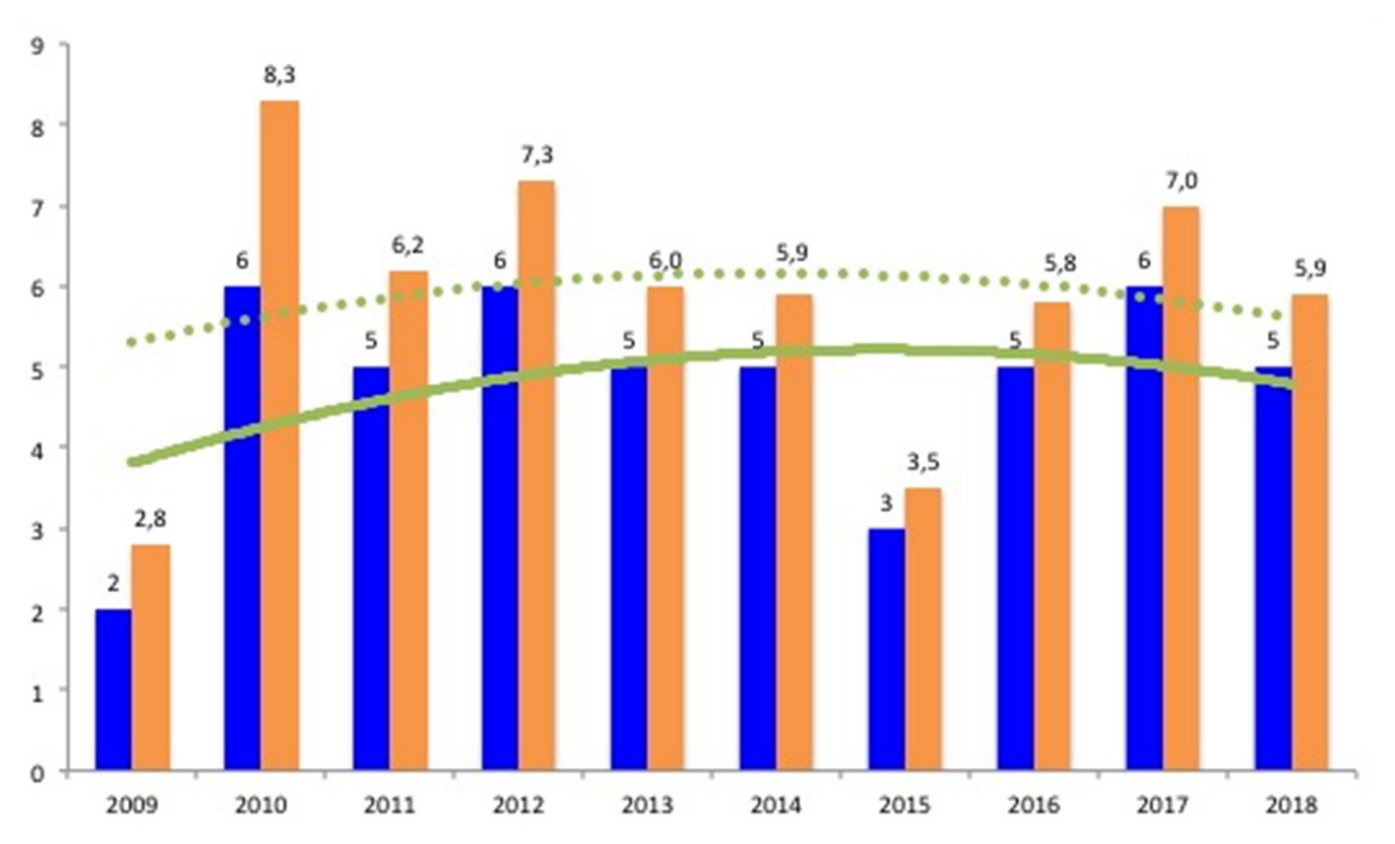
Acute rheumatic fever cases sorted by year with relative incidence (number of cases/100,000/year) among children resident in the province of Monza-Brianza. Blue bars: number of acute rheumatic fever (ARF) cases for each year. Orange bars: annual incidence of ARF/100,000. Continuous and dashed lines reflect trends for annual cases and incidence, respectively.

In order to calculate the annual incidence of ARF, only children resident in the Province of Monza-Brianza were included in this part of the analysis. Of the remaining 49 children, 2 were excluded because of their age (both 4 years old). Therefore, over a 10-year time period, 47 patients aged between 5 and 14 years were identified. The geographical distribution of ARF cases sorted by patient's residence is shown in Figure 1. The median incidence during the study time was 5.7/100,000 (range, 2.8–8.3/100,000) (Table 2). The number of cases for each year did not show an upward trend over 10 years, as well as the annual incidence per 100,000 school-age children (Figure 2).

**Table 2:** Cases of acute rheumatic fever (ARF) among children (5–14 of age) of the Province of Monza-Brianza.

**Year**	**No. of cases**	**Age-matched population**	**No. of cases per 100,000**
2009	2	72,104	2.8
2010	6	72,679	8.3
2011	5	80,507	6.2
2012	6	82,063	7.3
2013	5	83,770	6.0
2014	5	84,758	5.9
2015	3	85,259	3.5
2016	5	85,576	5.8
2017	6	85,826	7.0
2018	5	85,430	5.9

## Discussion

The improvements in living conditions and hygiene along with the increased accessibility to the healthcare system were the main reasons for the decline of ARF incidence worldwide during the nineteenth century, together with the increased use of antibiotics, especially in industrialized countries.

Nowadays, globalization might have altered this trend. Indeed, migratory flows have contributed to the resurgence of several diseases such as those related to infectious agents.

To date, updated epidemiological studies on ARF incidence in developed countries are scanty. Furthermore, the 2015 revision of Jones criteria makes the need for up-to-date information on ARF incidence more compelling.

Herein, we described ARF features over a 10-year-period in an area with a high population density such as the Province of Monza-Brianza, Lombardy, Italy. Heart involvement and arthritis were the most common manifestations, either isolated or in association; however, chorea was not rare. The median incidence of ARF among school-aged children residents in this province of northern Italy between 2009 and 2018 was 5.7/100,000 (range, 2.8–8.3/100,000), configuring children living in this area as a moderate–high-risk population for ARF. However, it has to be noted that the annual incidence of ARF depends not only on the number of ARF cases per year but also on the number of school-aged children (5–14 years) of the same year in the area. In this regard, we observed an upward trend of the school-aged population in the Province of Monza-Brianza (by 13,000 over 10 years) (Table 2). Overall, the annual incidence of ARF was constantly above the cutoff point of low-risk population (2/100,000); nevertheless, no upward trend was documented.

The age at onset and the slightly male prevalence observed in our study are comparable to previous similar studies (1, 7, 17, 18). Carditis and arthritis were the predominant features of ARF in our cohort, as frequently observed. The rate of carditis in our cohort was similar to what was reported by Breda et al. (1) and Fabi et al. (18) (49 and 57%, respectively), whereas Grassi et al. (7) documented a higher incidence of valvulitis (76%). However, we reported a lower rate of arthritis compared to other studies (39 vs. 53–66% of other studies) (1, 7, 18). This might be partially explained by the high rate of chorea observed in our population (29 vs. 5.7–21% of other studies), giving the subacute nature of this manifestation in ARF (1, 7, 18). The same assumption might be postulated for the lower rate of increased inflammatory markers compared to other studied (1, 7, 18).

With regard to ARF treatment, the majority received ibuprofen, confirming the clinicians' habit to avoid aspirin in children, given its safety profile. Corticosteroids were prescribed more frequently to patients with carditis, as expected. Furthermore, in agreement with recent findings (19), the majority of patients with chorea received corticosteroids.

The burden of the recommended GAS secondary prophylaxis in children is still concerning. After several years of continuous prophylaxis, family and children often prefer to discontinue these injections despite being suggested otherwise.

In this multicentric cohort, the recommended duration of GAS secondary prophylaxis was predominantly until 21 years of age, although some patients, even with carditis, were recommended to discontinue the prophylaxis before. Conversely, the frequency of administrations varied between 3 and 4 weeks according to each center. This highlights the lack of uniformity among participating centers that might be secondary to scarce knowledge of up-to-date information related to secondary prophylaxis and to the epidemiological status of our region.

A recent multicentric retrospective study showed that a significant number of children with ARF had a low adherence to secondary prophylaxis (20, 21). Nevertheless, the prophylaxis adherence was not associated with ARF recurrences or RDH (20, 21). In this regard, updated trials on GAS secondary prophylaxis in ARF are advisable since the evidence of current recommendations dates back 50 years (22–24).

So far, available updated data on ARF rates in Italy are scarce and difficult to compare given the different methodologies used. There is no national registry for ARF. Up-to-date information about ARF incidence is abstracted by monocentric experience or regional studies.

Breda et al. (1) conducted an epidemiological study between 2000 and 2009 in a region of Central Italy (Abruzzo). The overall incidence rate of ARF was 4.1/100,000 (range, 2.26–5.58/100,000) among children aged 0–18 years with a significant increase in the incidence of about 10% during the observation time (1).

Licciardi et al. (17) reported the incidence of ARF among children (age range, 5–14 years) between 2007 and 2016 in the Northwest of Italy (Turin). They documented a rate of ARF steadily above the threshold of low-risk population ranging between 3.2 and 9.6 per 100,000. Similar to our findings, they did not identify an increasing trend of ARF cases over the study time (17).

Recently, Munteanu et al. reported the rate of hospitalization of ARF in Lombardy by analyzing hospital discharge records between 2014 and 2016 (24). They found an overall rate of hospitalization of 4.24 cases per 100,000 children and adolescents (<18 years). In contrast with our findings, they reported an upward trend in ARF cases. However, the same authors stated that the short duration of the study observation in their cohort might have influenced the results as well as the possibility that some ARF cases reported in this study are not incident cases but prevalent ones (25). Our study presents several limitations that are in part intrinsic in its retrospective design. The lack of information about the follow-up of these patients is one limitation. Furthermore, multicentric studies, despite benefitting of larger numbers, are more prone to biases due to the lack of uniformity in clinical approach and data reporting.

In conclusion, we reported principal features of ARF patients between 2009 and 2018 in the Province of Monza-Brianza, a highly populated area of Northern Italy. We found an incidence rate of ARF among children aged 5–14 years constantly above the threshold of low-risk area as defined in the 2015 revision of Jones criteria. Therefore, the diagnosis of ARF should be based on the moderate–high-risk set of Jones criteria. However, given the burden of secondary prophylaxis, expert opinion is advisable when the diagnosis of ARF is uncertain.

## Data Availability Statement

The raw data supporting the conclusions of this article will be made available by the authors, without undue reservation.

## Ethics Statement

Ethical review and approval was not required for the study on human participants in accordance with the local legislation and institutional requirements. Written informed consent from the participants' legal guardian/next of kin was not required to participate in this study in accordance with the national legislation and the institutional requirements.

## Author Contributions

AM conceptualized and designed the study, drafted the initial manuscript, and reviewed and revised the manuscript. AB, GT, LM, MS, PC, FM, FC, RB, MA, AC, and CA designed the data collection instruments and collected data. RC, TV, and MP conceptualized and designed the study, coordinated and supervised data collection, and critically reviewed the manuscript for important intellectual content. All authors approved the final manuscript as submitted and agree to be accountable for all aspects of the work.

## Conflict of Interest

The open access publication fee was covered by PEDIATRICA SPECIALIST srl. The authors declare that the research was conducted in the absence of any commercial or financial relationships that could be construed as a potential conflict of interest.

## References

[1] Breda L Marzetti V Gaspari S Del Torto M Chiarelli F Altobelli E. Population-based study of incidence and clinical characteristics of rheumatic fever in Abruzzo, central Italy, 2000-2009. J Pediatr. (2012) 160:832–6.e1. 10.1016/j.jpeds.2011.10.009 22104560

[2] Siegel AC Johnston E Stollerman GH. Controlled studies of streptococcal pharyngitis in a pediatric population, I: factors related to attack rates of rheumatic fever. N Engl J Med. (1961) 265:559–66. 10.1056/NEJM196109212651201

[3] Sandson J Hamerman D Janis R Rojkind M. Immunologic and chemical similarities between the streptococcus and human connective tissue. Trans Assoc Am Physicians. (1968) 81:249–57. 4976488

[4] Tomai M Kotb M Majumdar G Beachey EH. Superantigenicity of streptococcal M protein. J Exp Med. (1990) 172:359–62. 10.1084/jem.172.1.359 2358781 PMC2188171

[5] Engel ME Stander R Vogel J Adeyemo AA Mayosi BM. Genetic susceptibility to acute rheumatic fever: a systematic review and meta-analysis of twin studies. PLoS ONE. (2011) 6:e25326. 10.1371/journal.pone.0025326 21980428 PMC3184125

[6] Bryant PA Robins-Browne R Carapetis JR Curtis N. Some of the people, some of the time: susceptibility to acute rheumatic fever. Circulation. (2009) 119:742–53. 10.1161/CIRCULATIONAHA.108.792135 19204317

[7] Grassi A Fesslovà V Carnelli V Boati E Dell'era L Salice P. Clinical characteristics and cardiac outcome of acute rheumatic fever in Italy in the last 15 years. Clin Exp Rheumatol. (2009) 27:366–72. 19473584

[8] Watkins DA Roth GA. Global burden of rheumatic heart disease. N Engl J Med. (2018) 378:e2. 10.1056/NEJMc1714503 29298146

[9] Carapetis JR Steer AC Mulholland EK Weber M. The global burden of group a streptococcal diseases. Lancet Infect Dis. (2005) 5:685–94. 10.1016/S1473-3099(05)70267-X 16253886

[10] Tibazarwa K Volmink J Mayosi B. Incidence of acute rheumatic fever in the world: a systematic review of population-based studies. Heart. (2008) 94:1534–40. 10.1136/hrt.2007.141309 18669552

[11] Karthikeyan G Guilherme L. Acute rheumatic fever. Lancet. (2018) 392:161–74. 10.1016/S0140-6736(18)30999-1 30025809

[12] Osborne W Sable C Scheel A Zühlke L Watkins D Beaton A. Trends and presentation patterns of acute rheumatic fever hospitalisations in the United States. Cardiol Young. (2019) 29:1387–90. 10.1017/S1047951119002270 31571555

[13] Jones T. The diagnosis of rheumatic fever. JAMA. (1944) 126:481–84. 10.1001/jama.1944.02850430015005

[14] Dajani AS Ayoub E Bierman FZ Bisno AL Denny FW Durack DT. Special writing group of the committee on rheumatic fever, endocarditis, and kawasaki disease of the council on cardiovascular disease in the young of the American Heart Association. Guidelines for the diagnosis of rheumatic fever: Jones criteria, 1992 update. JAMA. (1992) 268:2069–73. 10.1001/jama.268.15.2069 1404745

[15] Gewitz MH Baltimore RS Tani LY Sable CA Shulman ST Carapetis J. American Heart Association Committee on rheumatic fever, endocarditis, and kawasaki disease of the council on cardiovascular disease in the young. Revision of the Jones Criteria for the diagnosis of acute rheumatic fever in the era of Doppler echocardiography: a scientific statement from the American Heart Association. Circulation. (2015) 131:1806–18. 10.1161/CIR.0000000000000205 25908771

[16] Pastore S De Cunto A Benettoni A Berton E Taddio A Lepore L. The resurgence of rheumatic fever in a developed country area: the role of echocardiography. Rheumatology. (2011) 50:396–400. 10.1093/rheumatology/keq290 21047802

[17] Licciardi F Scaioli G Mulatero R Marolda A Delle Piane M Martino S. Epidemiologic impact of the new guidelines for the diagnosis of acute rheumatic fever. J Pediatr. (2018) 198:25–8.e1. 10.1016/j.jpeds.2018.02.024 29605389

[18] Fabi M Calicchia M Miniaci A Balducci A Tronconi E Bonetti S. Carditis in acute rheumatic fever in a high-income and moderate-risk country. J Pediatr. (2019) 215:187–91. 10.1016/j.jpeds.2019.07.072 31587860

[19] Favaretto E Gortani G Simonini G Pastore S Di Mascio A Cimaz R. Preliminary data on prednisone effectiveness in children with Sydenham chorea. Eur J Pediatr. (2020) 179:993–7. 10.1007/s00431-020-03574-y 31965299

[20] Taddio A Pillon R Pastore S Monasta L Tommasini A Di Battista C. Italian paediatric rheumatology study group. acute rheumatic fever prophylaxis in high-income countries: clinical observations from an Italian multicentre, retrospective study. Clin Exp Rheumatol. (2020) 38:1016–20. 31969217

[21] Amarilyo G Chodick G Zalcman J Koren G Levinsky Y Somekh I. Poor long-term adherence to secondary penicillin prophylaxis in children with history of rheumatic fever. Semin Arthritis Rheum. (2019) 48:1019–24. 10.1016/j.semarthrit.2018.10.015 30415945

[22] Feinstein AR Wood HF Epstein JA Taranta A Simpson R Tursky E. A controlled study of three methods of prophylaxis against streptococcal infection in a population of rheumatic children. II. Results of the first three years of the study, including methods for evaluating the maintenance of oral prophylaxis. N Engl J Med. (1959) 260:697–702. 10.1056/NEJM195904022601405 13644570

[23] Kohm KH Milzed A Maclean H. Prophylaxis of recurrences of rheumatic fever with penicillin given orally; final report of a five year study. J Am Med Assoc. (1953) 151:347–51. 13010995

[24] Spector AJ. Measurement of the incidence of acute rheumatic fever: a methodological study. Am J Public Health Nations Health. (1968) 58:1950–64. 10.2105/AJPH.58.10.1950 5693020 PMC1228960

[25] Munteanu V Petaccia A Contecaru N Amodio E Agostoni CV. Paediatric acute rheumatic fever in developed countries: neglected or negligible disease? Results from an observational study in Lombardy (Italy). AIMS Public Health. (2018) 5:135–43. 10.3934/publichealth.2018.2.135 30094276 PMC6079050

